# Crystal structure and Hirshfeld surface analysis of 2-amino-5-{(1*E*)-1-[(carbamo­thioyl­amino)­imino]eth­yl}-4-methyl-1,3-thia­zol-3-ium chloride monohydrate

**DOI:** 10.1107/S2056989023007090

**Published:** 2023-08-17

**Authors:** Elnur Z. Huseynov, Mehmet Akkurt, Ivan Brito, Ajaya Bhattarai, Farid N. Naghiyev, Khammed A. Asadov, Abel M. Maharramov

**Affiliations:** a Department of Chemistry, Baku State University, Z. Khalilov str. 23, Az, 1148 Baku, Azerbaijan; b Department of Physics, Faculty of Sciences, Erciyes University, 38039 Kayseri, Türkiye; c Departamento de Química, Facultad de Ciencias Básicas, Universidad de Antofagasta, Avenida Angamos 601, Casilla 170, Antofagasta 1240000, Chile; d Department of Chemistry, M.M.A.M.C (Tribhuvan University) Biratnagar, Nepal; Vienna University of Technology, Austria

**Keywords:** crystal structure, 1,3-thia­zol-3-ium, hydrogen bonds, hydrogen-bonded network, Hirshfeld surface analysis

## Abstract

In the hydrated title salt, C_7_H_12_N_5_S_2_^+^·Cl^−^·H_2_O, the asymmetric unit comprises one 2-amino-5-{(1*E*)-1-[(carbamo­thioyl­amino)­imino]­eth­yl}-4-methyl-1,3-thia­zol-3-ium cation, one chloride anion and one water mol­ecule of crystallization. The cation is nearly flat (r.m.s. deviation of non-H atoms is 0.0814 Å), with the largest deviation of 0.1484 (14) Å observed for one of the methyl C atoms. In the crystal, the cations are linked by O—H⋯Cl, N—H⋯Cl, N—H⋯O, N—H⋯S and C—H⋯S hydrogen bonds, forming a tri-periodic network. The most important contributions to the crystal packing are from H⋯H (35.4%), S⋯H/H⋯S (24.4%), N⋯H/H⋯N (8.7%), Cl⋯H/H⋯Cl (8.2%) and C⋯H/H⋯C (7.7%) inter­actions.

## Abstract 2

In the crystal, the 2-amino-5-{(1*E*)-1-[(carbamo­thioyl­amino)­imino]­eth­yl}-4-methyl-1,3-thia­zol-3-ium cations are linked by O—H⋯Cl, N—H⋯Cl, N—H⋯O, N—H⋯S and C—H⋯S hydrogen bonds, forming a tri-periodic network.

## 1. Chemical context

**Figure 1:**
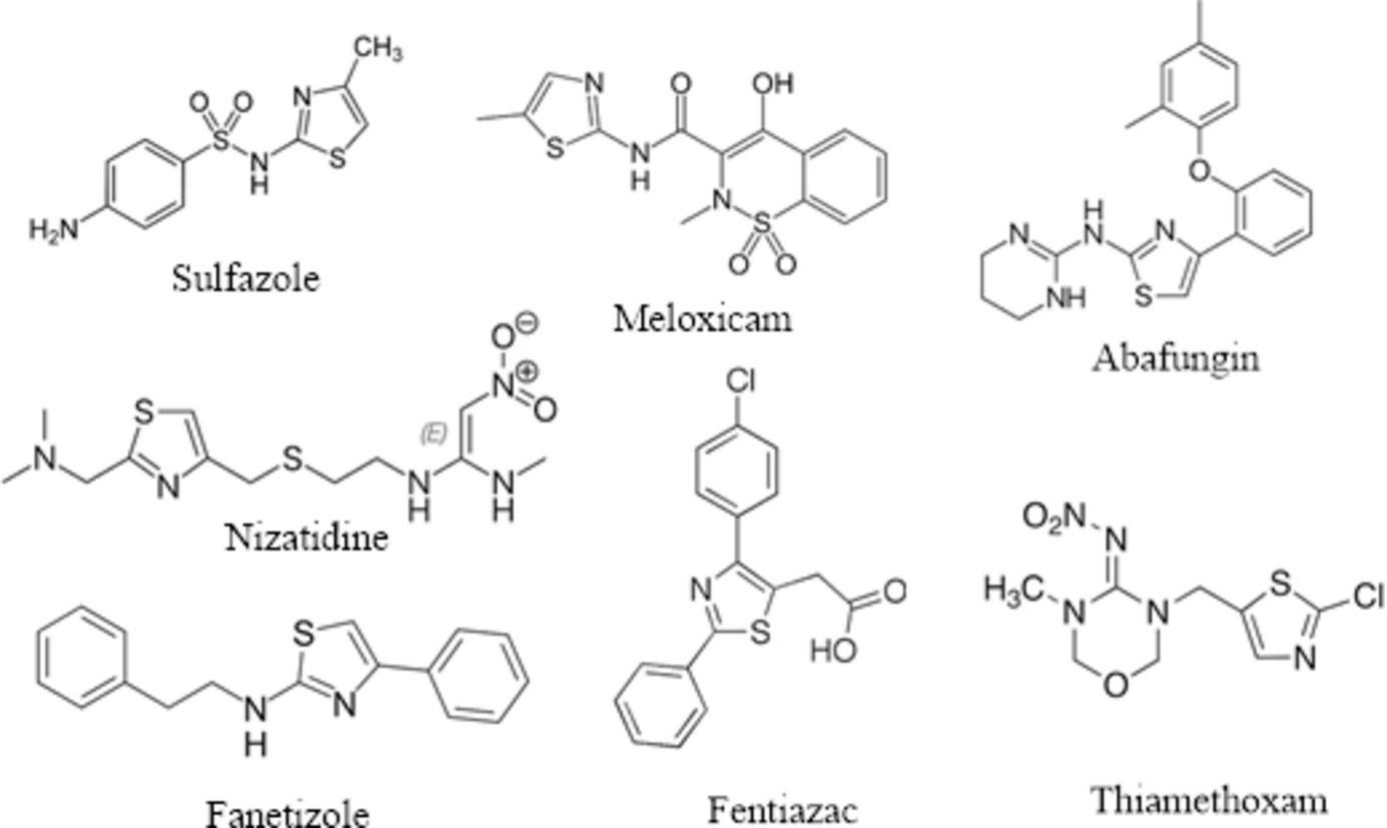
Chemical diagrams of some thia­zole-containing marketed drugs with trade names.

Heterocyclic systems account for many important organic compounds (Maharramov *et al.*, 2011*b* ▸; Abdelhamid *et al.*, 2014 ▸). In particular, five- and six-membered heterocycles are applied in different branches of chemistry, including sustainable chemistry (Montes *et al.*, 2018 ▸), drug design and development (Khalilov *et al.*, 2021 ▸; Tas *et al.*, 2023 ▸) or material science (Yin *et al.*, 2020 ▸). The thia­zole core is one of the most common five-membered heteroaromatic ring systems (Yadigarov *et al.*, 2009 ▸; Khalilov, 2021 ▸). Thia­zoles have potent biological applications and represent an essential core scaffold present in many natural (thi­amine, penicillin) and synthetic medicinally important compounds (Chhabria *et al.*, 2016 ▸), such as sulfazole, ritonavir, abafungin, fanetizole, meloxicam, fenti­azac, nizatidine and thia­methoxam (Fig. 1 ▸). A variety of thia­zole derivatives are also used as target products as well as synthetic inter­mediates (Maharramov *et al.*, 2011*a* ▸; Kekeçmuhammed *et al.*, 2022 ▸). 
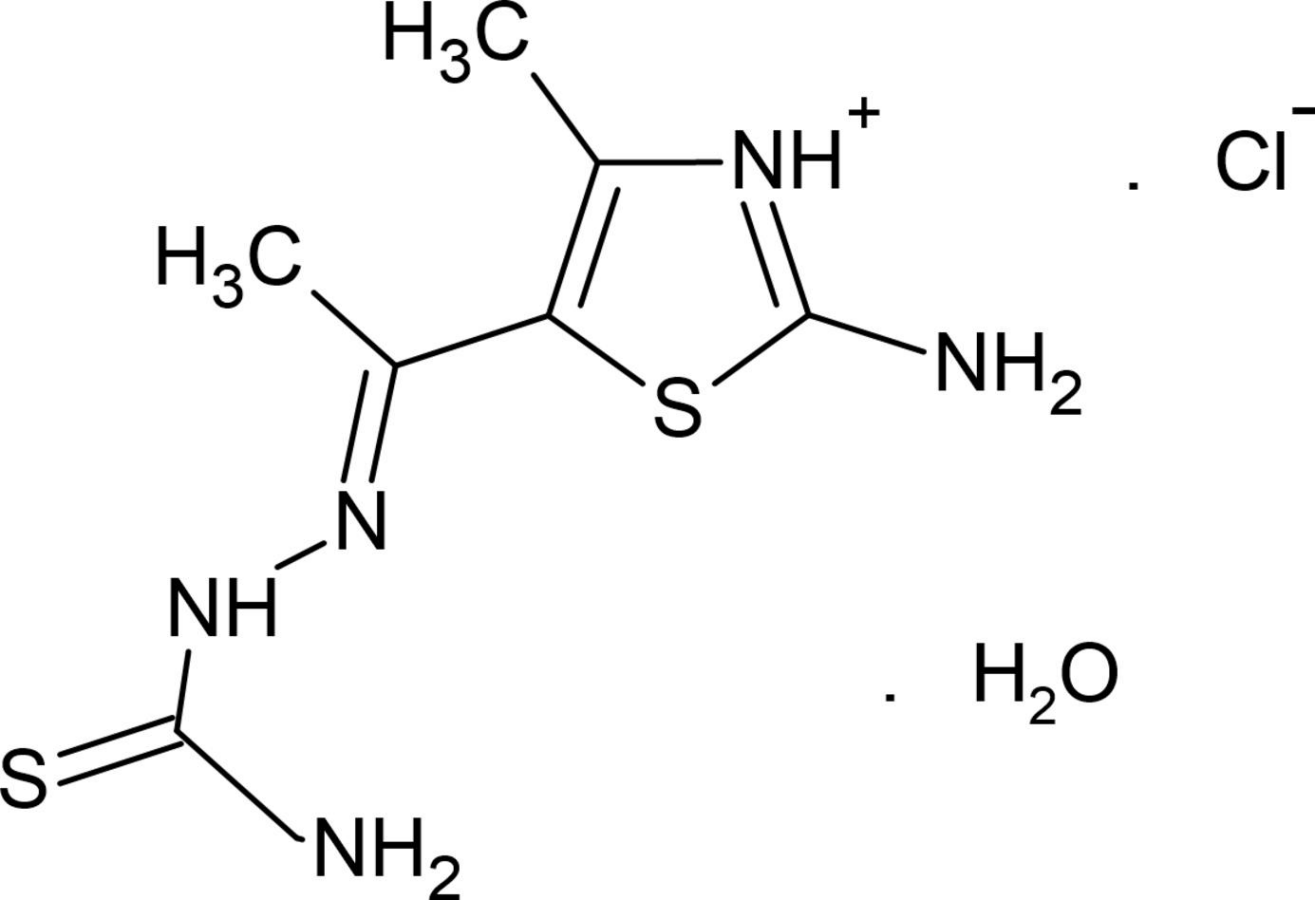


In a continuation of our structural investigations of heterocyclic systems associated with biological activities (Akkurt *et al.*, 2018 ▸; Askerov *et al.*, 2020 ▸; Karimli *et al.*, 2023 ▸), we report here the crystal structure and Hirshfeld surface analysis of the hydrated title salt, C_7_H_12_N_5_S_2_^+^·Cl^−^·H_2_O, (I).

## 2. Structural commentary

**Figure 2:**
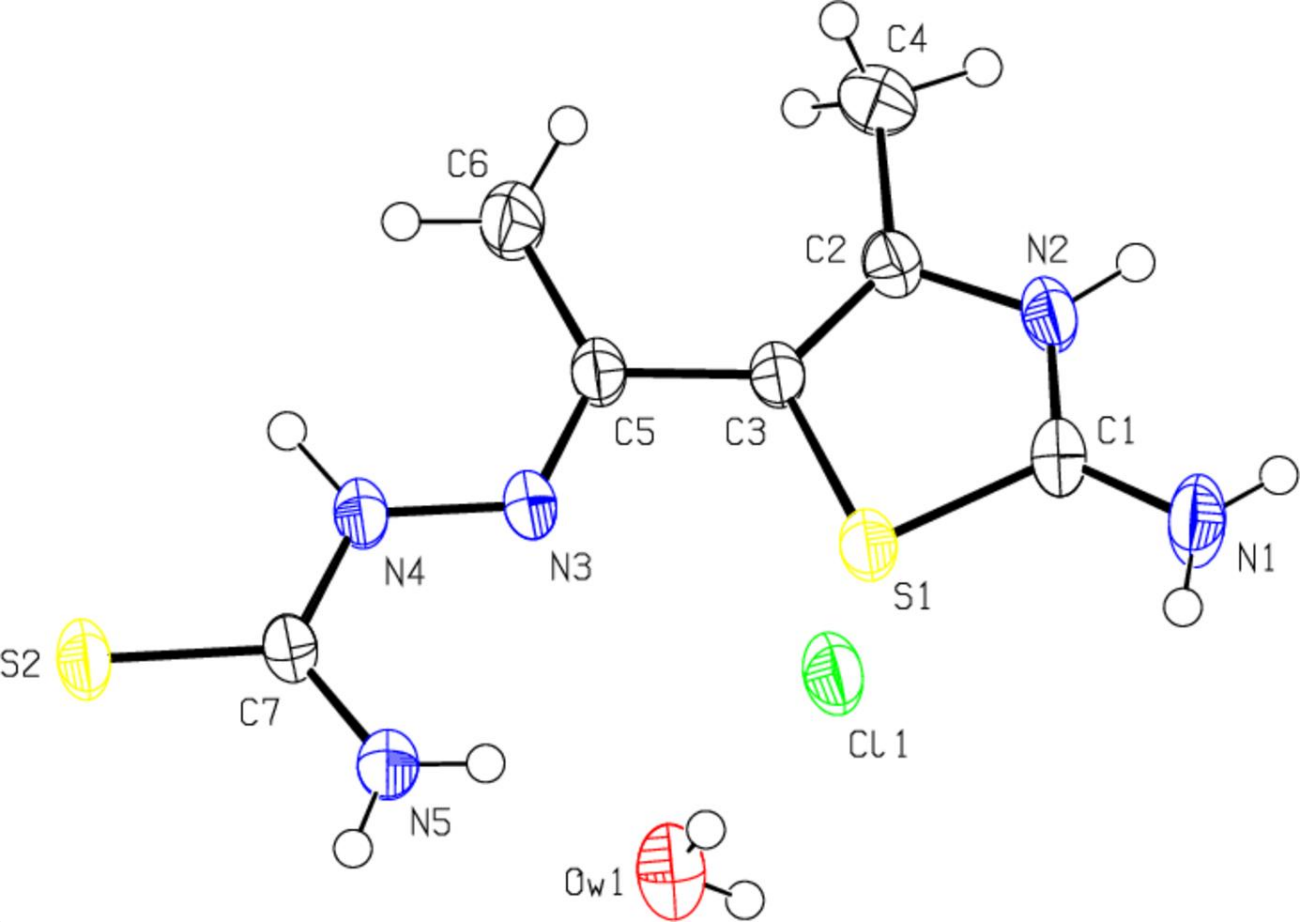
The mol­ecular structure of (I), showing the atom labeling and displacement ellipsoids drawn at the 50% probability level.

The asymmetric unit of (I) (Fig. 2 ▸) comprises one 2-amino-5-{(1*E*)-1-[(carbamo­thioyl­amino)­imino]­eth­yl}-4-methyl-1,3-thia­zol-3-ium cation, C_7_H_12_N_5_S_2_^+^, one chloride anion and one water mol­ecule of crystallization. In the 1,3-thia­zol-3-ium ring, as expected, the C1—N2 distance of 1.3309 (16) Å indicates double-bond character, while the C2—N2 distance of 1.3885 (14) Å has more single-bond character.

In the amino-*N*′-[(1*Z*)-ethyl­idene]ethane­thio­hydrazide group, the S2—C7—N4—N3, N5—C7—N4—N3, C7—N4—N3—C5 and N4—N3—C5—C6 torsion angles are 178.17 (8), −0.63 (16), 174.48 (10) and 0.16 (18)°, respectively. The title compound shows bond lengths and angles that are typical and are in agreement with those reported for the related compounds discussed in the *Database survey* section.

The cation is nearly flat (r.m.s. deviation of the 14 non-H atoms is 0.0814 Å), with the largest deviations observed for C6 [0.1484 (14) Å], N1 [0.1357 (10) Å], and S2 [0.1399 (6) Å].

## 3. Supra­molecular features and Hirshfeld surface analysis

**Figure 3:**
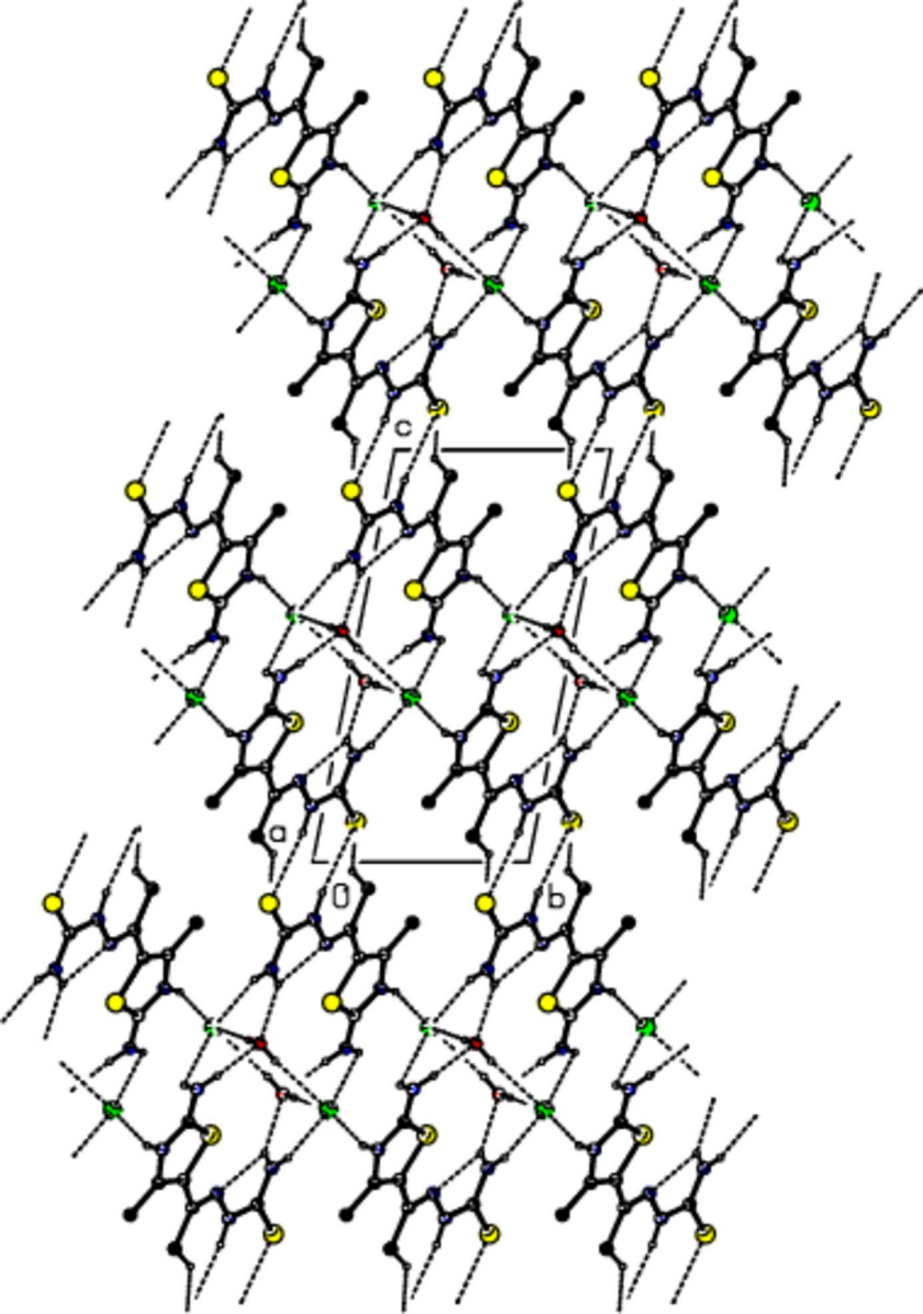
View of the packing of (I) along the *a* axis with the O—H⋯Cl, N—H⋯Cl, N—H⋯O, N—H⋯S and C—H⋯S hydrogen bonds (dashed lines). H atoms not involved in hydrogen bonding have been omitted for clarity.

In the crystal of (I), the cations are linked by O—H⋯Cl, N—H⋯Cl, N—H⋯O, N—H⋯S and C—H⋯S hydrogen bonds (Table 1 ▸), forming a tri-periodic network (Figs. 3 ▸–5 ▸ ▸). Significant C—H⋯π or π–π inter­actions are not developed.

**Figure 6:**
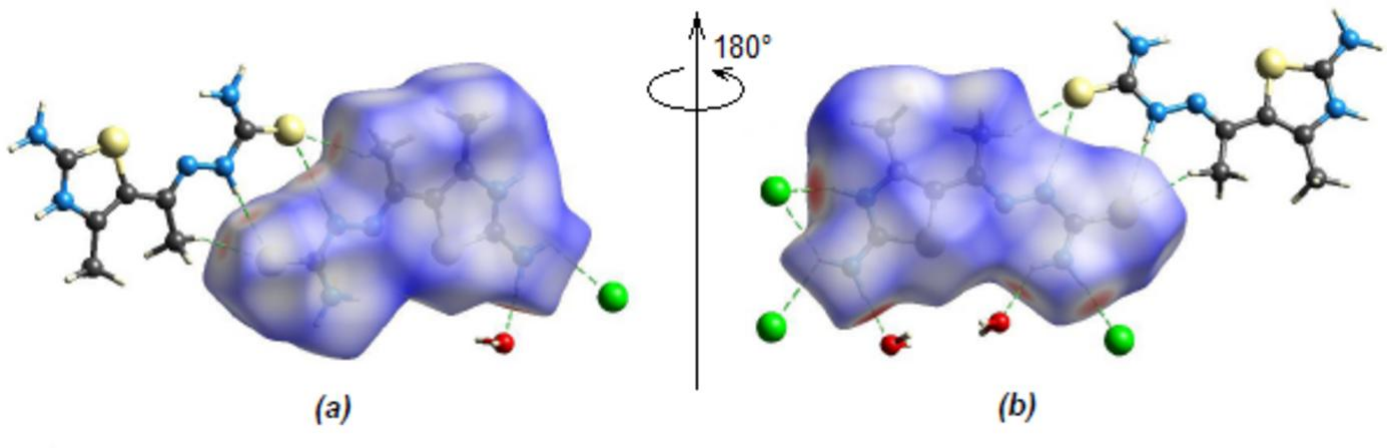
(*a*) Front and (*b*) back sides of the three-dimensional Hirshfeld surface of (I) mapped over *d*_norm_.

In order to visualize and qu­antify inter­molecular inter­actions (Table 2 ▸) in (I), a Hirshfeld surface analysis was performed using *Crystal Explorer 17.5* (Spackman *et al.*, 2021 ▸), which was also used for generation of the associated two-dimensional fingerprint plots. The Hirshfeld surface mapped over *d*_norm_ shows the inter­molecular contacts as red-colored spots, which indicate the O—H⋯Cl, N—H⋯Cl, N—H⋯O, N—H⋯S and C—H⋯S hydrogen bonds (Fig. 6 ▸).

**Figure 7:**
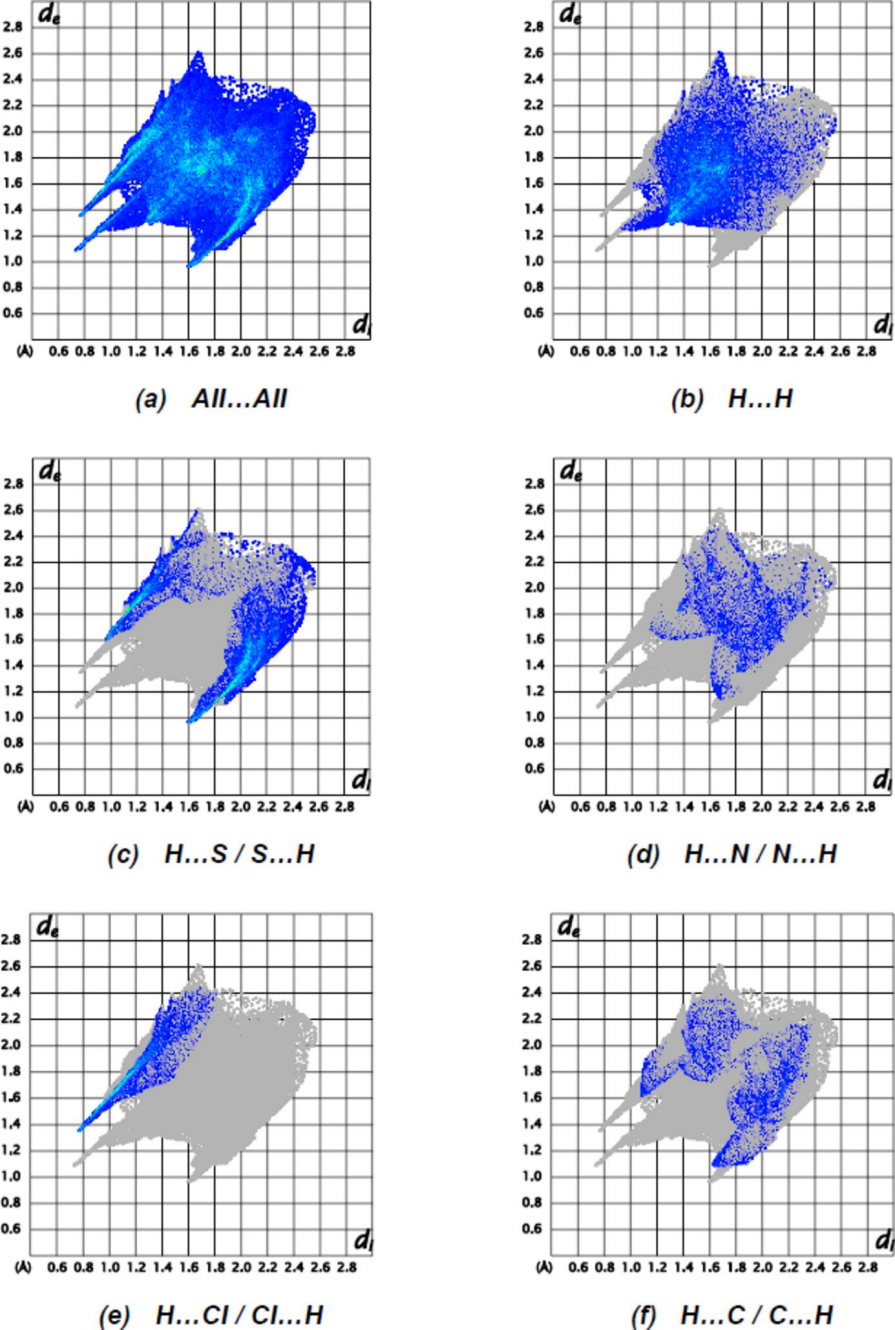
The two-dimensional fingerprint plots of (I), showing (*a*) all inter­actions, and delineated into (*b*) H⋯H, (*c*) S⋯H/H⋯S, (*d*) N⋯H/H⋯N, (*e*) Cl⋯H/H⋯Cl and (*f*) C⋯H/H⋯C inter­actions. [*d*_e_ and *d*_i_ represent the distances from a point on the Hirshfeld surface to the nearest atoms outside (external) and inside (inter­nal) the surface, respectively].

The two-dimensional fingerprint plots of the most abundant contacts are presented in Fig. 7 ▸. H⋯H (35.4%) and S⋯H/H⋯S (24.4%) contacts are responsible for the largest contributions to the Hirshfeld surface. Besides these contacts, N⋯H/H⋯N (8.7%), Cl⋯H/H⋯Cl (8.2%) and C⋯H/H⋯C (7.7%) inter­actions contribute significantly to the total Hirshfeld surface. The contributions of further contacts are only minor and amount to C⋯H/H⋯C (4.5%), S⋯C/C⋯S (2.4%), N⋯C/C⋯N (2.1%), N⋯N (1.9%), C⋯C (1.6%), S⋯N/N⋯S (1.3%), Cl⋯S/S⋯Cl (0.6%), Cl⋯C/C⋯Cl (0.6%), S⋯S (0.4%), N⋯O/O⋯N (%0.1) and S⋯O/O⋯S (0.1%).

## 4. Database survey

A search of the Cambridge Crystallographic Database (updated 20 March 2023; Groom *et al.*, 2016 ▸) using the 1,3-thia­zol-3-ium moiety as the search fragment revealed four closely related compounds: 2-anilino-3-(2-hy­droxy­prop­yl)-4-methyl-1,3-thia­zol-3-ium chloride (II) (Mohamed *et al.*, 2012 ▸), 2-amino-5-butyl-4-methyl-1,3-thia­zol-3-ium nitrate (III) (Zarychta *et al.*, 2003 ▸), 2-(2-thioxo-1,3-thia­zolidin-3-yl)-4,5-di­hydro-l,3-thia­zol-l-ium chloride (IV) (Raper *et al.*, 1996 ▸) and 2-ureido-1,3-thia­zol-3-ium di­hydrogen phosphate (V) (Gubina *et al.*, 2011 ▸).

In the crystal of (II), mol­ecules are linked by O—H⋯Cl and N—H⋯Cl hydrogen bonds, forming zigzag chains along [001]. There is also a C—H⋯Cl inter­action present. The crystal structure of (III) comprises a substituted thia­zolium ring that is connected to a nitrate ion *via* N—H⋯O hydrogen-bonding inter­actions. In the crystal of (IV), the mol­ecular packing is determined by inter­ionic N—H⋯Cl contacts. In the crystal of (V), the mol­ecules of substituted urea are connected by O—H⋯O hydrogen bonds into sheets. In turn, these sheets are connected to each other *via* N—H⋯O hydrogen bonds with hydrogen phosphate anions, forming a tri-periodic network.

## 5. Synthesis and crystallization

The title compound was synthesized using a reported procedure (Gomha *et al.*, 2016 ▸). Colorless crystals were obtained upon recrystallization from an ethanol/water (3:1 *v*:*v*) solution at room temperature

## 6. Refinement

Crystal data, data collection and structure refinement details are summarized in Table 3 ▸. The H atoms of the methyl groups were positioned geometrically and refined as riding with C—H = 0.96 Å, with *U*_iso_(H) = 1.5*U*_eq_(C). The H atoms attached to the N atom and the H atoms of the water mol­ecule were found in a difference-Fourier map. Their positional parameters were refined freely while setting *U*_iso_(H) = 1.2*U*_eq_(N) and 1.5*U*_eq_(O).

## Supplementary Material

Crystal structure: contains datablock(s) I. DOI: 10.1107/S2056989023007090/wm5691sup1.cif

Structure factors: contains datablock(s) I. DOI: 10.1107/S2056989023007090/wm5691Isup2.hkl

Supporting information file. DOI: 10.1107/S2056989023007090/wm5691Isup3.cml

CCDC reference: 2288159

Additional supporting information:  crystallographic information; 3D view; checkCIF report

## Figures and Tables

**Figure 4:**
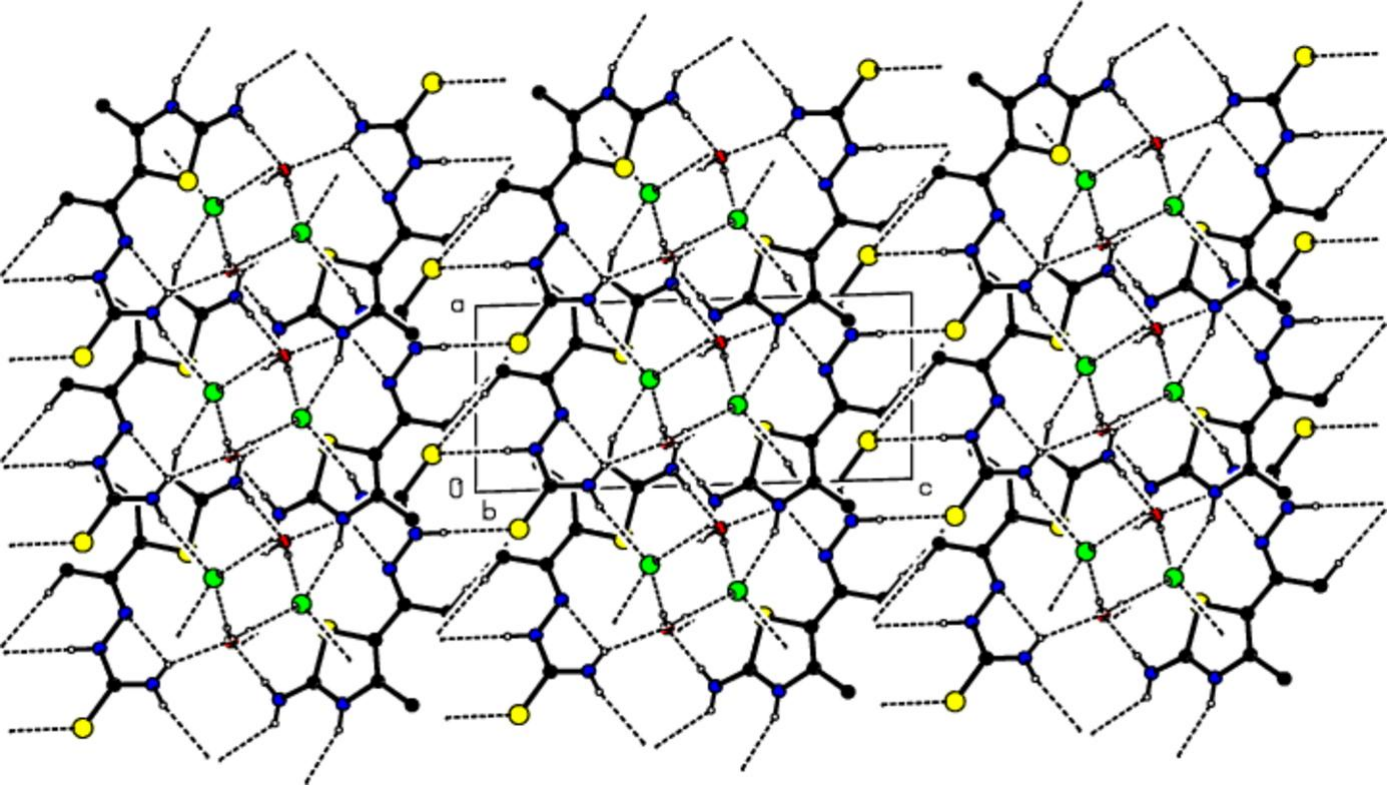
View of the same inter­actions as in Fig. 2 ▸ along the *b* axis.

**Figure 5:**
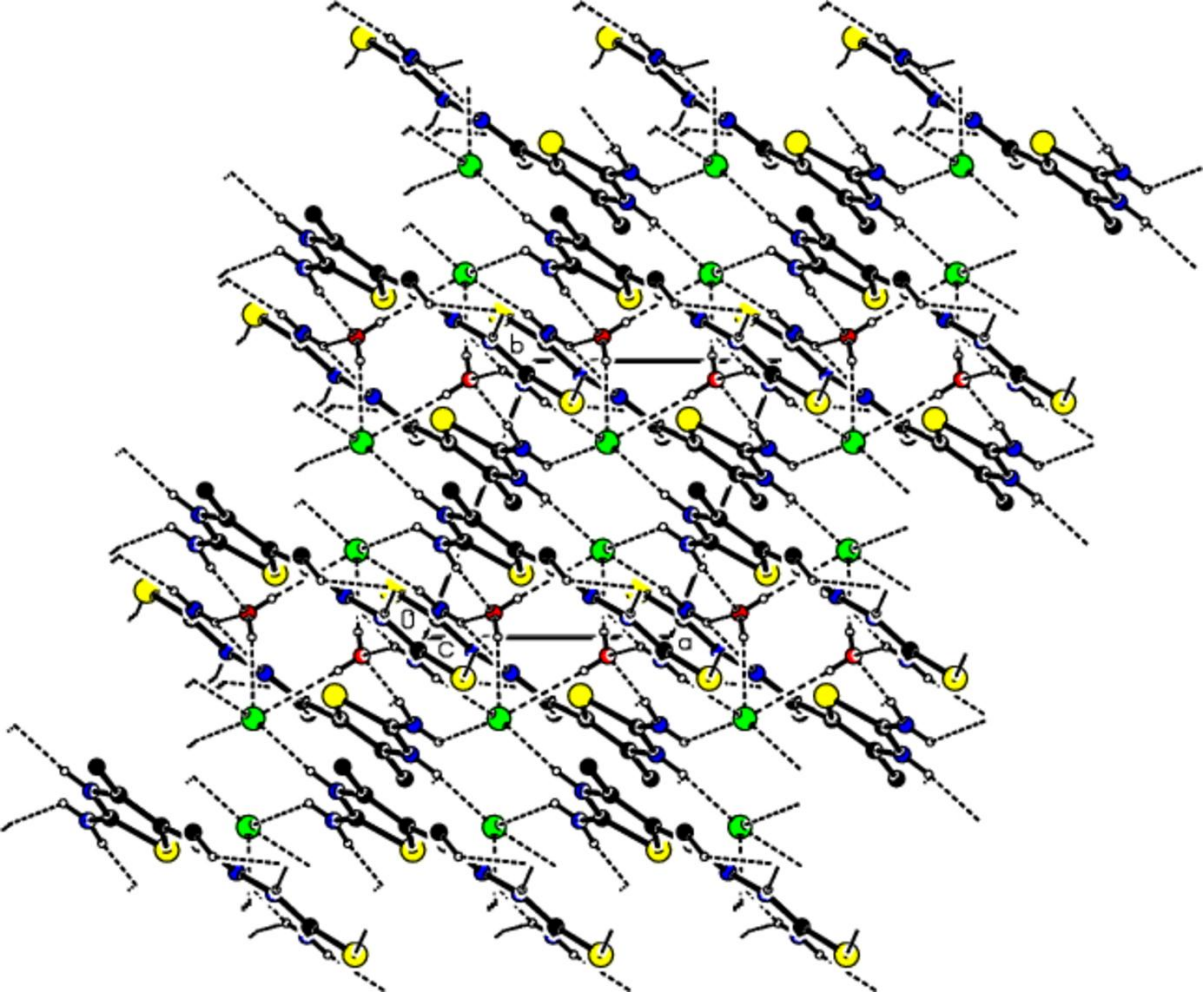
View of the same inter­actions as in Fig. 2 ▸ along the *c* axis.

**Table 1:** Hydrogen-bond geometry (Å, °)

*D*—H⋯*A*	*D*—H	H⋯*A*	*D*⋯*A*	*D*—H⋯*A*
O*W*1—H*W*1⋯Cl1^i^	0.79 (3)	2.45 (3)	3.2298 (13)	171 (3)
N2—H2⋯Cl1^ii^	0.853 (18)	2.277 (18)	3.0812 (11)	157.2 (15)
O*W*1—H*W*2⋯Cl1	0.71 (3)	2.50 (3)	3.2111 (14)	178 (3)
N1—H11⋯Cl1^iii^	0.78 (3)	2.81 (2)	3.2398 (15)	117.0 (18)
N1—H12⋯O*W*1^iv^	0.87 (2)	1.97 (2)	2.8354 (19)	174 (2)
N4—H41⋯S2^v^	0.943 (16)	2.686 (16)	3.6223 (11)	172.0 (14)
N5—H51⋯Cl1^vi^	0.81 (2)	2.54 (2)	3.3243 (13)	164.9 (18)
N5—H52⋯O*W*1^vii^	0.80 (2)	2.326 (19)	2.9899 (17)	141.2 (19)
C6—H6*C*⋯S2^v^	0.96	2.67	3.4923 (15)	143

Symmetry codes: (i) 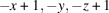 ; (ii) 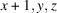 ; (iii) 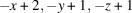 ; (iv) 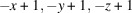 ; (v) 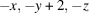 ; (vi) 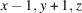 ; (vii) 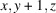 .

**Table 2:** Summary of short inter­atomic contacts (Å) in the title compound

Contact	Distance	Symmetry code
H52⋯O*W*1	2.33	*x*, 1 + *y*, *z*
C1⋯N5	3.37	1 + *x*, *y*, *z*
C7⋯H4*A*	2.83	−1 + *x*, 1 + *y*, *z*
H6*C*⋯S2	2.67	−*x*, 2 − *y*, −*z*
H4*C*⋯S2	3.08	1 − *x*, 2 − *y*, −*z*
H2⋯Cl1	2.28	1 + *x*, *y*, *z*
H11⋯Cl1	2.81	2 − *x*, 1 − *y*, 1 − *z*
H12⋯O*W*1	1.97	1 − *x*, 1 − *y*, 1 − *z*
N1⋯N1	3.27	2 − *x*, 1 − *y*, 1 − *z*
H51⋯Cl1	2.54	−1 + *x*, 1 + *y*, *z*
C6⋯C4	3.55	2 − *x*, 1 − *y*, −*z*
Cl1⋯H*W*2	2.50	*x*, *y*, *z*
Cl1⋯H*W*1	2.45	1 − *x*, −*y*, 1 − *z*

**Table 3:** Experimental details

Crystal data	
Chemical formula	C_7_H_12_N_5_S_2_^+^·Cl^−^·H_2_O
*M*_r_	283.80
Crystal system, space group	Triclinic, *P* 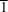
Temperature (K)	293
*a*, *b*, *c* (Å)	6.3279 (4), 7.7816 (6), 14.1342 (10)
α, β, γ (°)	77.191 (3), 83.660 (3), 67.860 (2)
*V* (Å^3^)	628.35 (8)
*Z*	2
Radiation type	Mo *K*α
μ (mm^−1^)	0.62
Crystal size (mm)	0.04 × 0.03 × 0.03

Data collection	
Diffractometer	Bruker APEXII CCD
Absorption correction	Multi-scan (*SADABS*; Krause *et al.*, 2015 ▸).
*T*_min_, *T*_max_	0.570, 0.747
No. of measured, independent and observed [*I* > 2σ(*I*)] reflections	34019, 6058, 4589
*R*_int_	0.053
(sin θ/λ)_max_ (Å^−1^)	0.833

Refinement	
*R*[*F* ^2^ > 2σ(*F* ^2^)], *wR*(*F* ^2^), *S*	0.036, 0.115, 1.05
No. of reflections	6058
No. of parameters	171
H-atom treatment	H atoms treated by a mixture of independent and constrained refinement
Δρ_max_, Δρ_min_ (e Å^−3^)	0.50, −0.29

Computer programs: *APEX2* (Bruker, 2016 ▸), *SAINT* (Bruker, 2016 ▸), *SHELXT* (Sheldrick, 2015*a* ▸), *SHELXL* (Sheldrick, 2015*b* ▸), *ORTEP-3 for Windows* (Farrugia, 2012 ▸) and *PLATON* (Spek, 2020 ▸).

## supplementary crystallographic information

### Crystal data

**Table:**

C_7_H_12_N_5_S_2_^+^·Cl^−^·H_2_O	*Z* = 2
*M**_r_* = 283.80	*F*(000) = 296
Triclinic, *P*1	*D*_x_ = 1.500 Mg m^−^^3^
*a* = 6.3279 (4) Å	Mo *K*α radiation, λ = 0.71073 Å
*b* = 7.7816 (6) Å	Cell parameters from 9921 reflections
*c* = 14.1342 (10) Å	θ = 3.0–36.3°
α = 77.191 (3)°	µ = 0.62 mm^−^^1^
β = 83.660 (3)°	*T* = 293 K
γ = 67.860 (2)°	Prism, colourless
*V* = 628.35 (8) Å^3^	0.04 × 0.03 × 0.03 mm

### Data collection

**Table:**

Bruker APEXII CCD diffractometer	4589 reflections with *I* > 2σ(*I*)
φ and ω scans	*R*_int_ = 0.053
Absorption correction: multi-scan (SADABS; Krause *et al.*, 2015).	θ_max_ = 36.3°, θ_min_ = 3.0°
*T*_min_ = 0.570, *T*_max_ = 0.747	*h* = −10→10
34019 measured reflections	*k* = −12→12
6058 independent reflections	*l* = −23→21

### Refinement

**Table:**

Refinement on *F*^2^	0 restraints
Least-squares matrix: full	Hydrogen site location: mixed
*R*[*F*^2^ > 2σ(*F*^2^)] = 0.036	H atoms treated by a mixture of independent and constrained refinement
*wR*(*F*^2^) = 0.115	*w* = 1/[σ^2^(*F*_o_^2^) + (0.0567*P*)^2^ + 0.0995*P*] where *P* = (*F*_o_^2^ + 2*F*_c_^2^)/3
*S* = 1.05	(Δ/σ)_max_ = 0.001
6058 reflections	Δρ_max_ = 0.50 e Å^−^^3^
171 parameters	Δρ_min_ = −0.29 e Å^−^^3^

### Special details

**Table:**

Geometry. All esds (except the esd in the dihedral angle between two l.s. planes) are estimated using the full covariance matrix. The cell esds are taken into account individually in the estimation of esds in distances, angles and torsion angles; correlations between esds in cell parameters are only used when they are defined by crystal symmetry. An approximate (isotropic) treatment of cell esds is used for estimating esds involving l.s. planes.

### Fractional atomic coordinates and isotropic or equivalent isotropic displacement parameters (Å^2^)

**Table:**

	*x*	*y*	*z*	*U*_iso_*/*U*_eq_	
C1	1.00052 (18)	0.66580 (16)	0.37114 (8)	0.0336 (2)	
C2	1.00425 (17)	0.58328 (15)	0.22387 (8)	0.03023 (18)	
C3	0.78120 (16)	0.69558 (14)	0.23070 (7)	0.02811 (17)	
C4	1.1391 (2)	0.4821 (2)	0.14588 (10)	0.0429 (3)	
H4A	1.053893	0.420248	0.123677	0.064*	
H4B	1.281001	0.389685	0.171192	0.064*	
H4C	1.169126	0.571545	0.092670	0.064*	
C5	0.59592 (16)	0.76248 (15)	0.16319 (7)	0.02893 (17)	
C6	0.6438 (2)	0.7189 (2)	0.06335 (10)	0.0474 (3)	
H6A	0.648883	0.593175	0.065682	0.071*	
H6B	0.788013	0.727617	0.039126	0.071*	
H6C	0.525339	0.807944	0.021177	0.071*	
C7	0.01732 (16)	1.05991 (15)	0.17123 (7)	0.02918 (17)	
N1	1.0867 (2)	0.6736 (2)	0.45014 (10)	0.0471 (3)	
H11	1.215 (4)	0.611 (3)	0.4604 (14)	0.057*	
H12	0.992 (4)	0.756 (3)	0.4827 (15)	0.057*	
N2	1.12182 (15)	0.56820 (14)	0.30449 (7)	0.03419 (18)	
H2	1.261 (3)	0.496 (2)	0.3136 (12)	0.041*	
N3	0.40159 (14)	0.86322 (14)	0.19705 (7)	0.03073 (16)	
N4	0.21575 (15)	0.94127 (14)	0.13782 (7)	0.03273 (18)	
H41	0.214 (3)	0.927 (2)	0.0734 (12)	0.039*	
N5	0.01285 (18)	1.09259 (17)	0.25930 (8)	0.0395 (2)	
H51	−0.101 (3)	1.160 (3)	0.2836 (13)	0.047*	
H52	0.117 (3)	1.046 (3)	0.2953 (13)	0.047*	
S1	0.71982 (4)	0.78020 (4)	0.33975 (2)	0.03514 (7)	
S2	−0.21036 (5)	1.16255 (5)	0.09918 (2)	0.03939 (8)	
Cl1	0.57747 (5)	0.30475 (5)	0.39936 (2)	0.04264 (8)	
OW1	0.23501 (19)	0.07771 (18)	0.43851 (9)	0.0517 (3)	
HW1	0.288 (4)	−0.009 (4)	0.4806 (19)	0.078*	
HW2	0.308 (4)	0.131 (4)	0.4302 (19)	0.078*	

### Atomic displacement parameters (Å^2^)

**Table:**

	*U*^11^	*U*^22^	*U*^33^	*U*^12^	*U*^13^	*U*^23^
C1	0.0290 (4)	0.0352 (5)	0.0351 (5)	−0.0092 (4)	−0.0118 (4)	−0.0030 (4)
C2	0.0241 (4)	0.0307 (4)	0.0325 (4)	−0.0064 (3)	−0.0040 (3)	−0.0042 (3)
C3	0.0230 (4)	0.0313 (4)	0.0283 (4)	−0.0065 (3)	−0.0052 (3)	−0.0062 (3)
C4	0.0341 (5)	0.0456 (6)	0.0429 (6)	−0.0052 (5)	0.0031 (4)	−0.0152 (5)
C5	0.0240 (4)	0.0324 (4)	0.0296 (4)	−0.0081 (3)	−0.0065 (3)	−0.0057 (3)
C6	0.0341 (5)	0.0638 (8)	0.0390 (6)	−0.0028 (5)	−0.0082 (4)	−0.0231 (6)
C7	0.0230 (4)	0.0327 (4)	0.0301 (4)	−0.0080 (3)	−0.0063 (3)	−0.0037 (3)
N1	0.0405 (5)	0.0552 (7)	0.0440 (6)	−0.0094 (5)	−0.0204 (4)	−0.0116 (5)
N2	0.0236 (3)	0.0361 (4)	0.0378 (4)	−0.0047 (3)	−0.0092 (3)	−0.0043 (3)
N3	0.0225 (3)	0.0370 (4)	0.0303 (4)	−0.0071 (3)	−0.0071 (3)	−0.0054 (3)
N4	0.0236 (3)	0.0403 (5)	0.0309 (4)	−0.0050 (3)	−0.0076 (3)	−0.0086 (3)
N5	0.0290 (4)	0.0525 (6)	0.0316 (4)	−0.0055 (4)	−0.0053 (3)	−0.0121 (4)
S1	0.02593 (11)	0.04268 (15)	0.03359 (13)	−0.00389 (10)	−0.00727 (9)	−0.01324 (11)
S2	0.02756 (12)	0.04546 (16)	0.03856 (14)	−0.00214 (10)	−0.01341 (10)	−0.00894 (12)
Cl1	0.02891 (12)	0.04757 (16)	0.04456 (16)	−0.00184 (10)	−0.01143 (10)	−0.01183 (12)
OW1	0.0426 (5)	0.0549 (6)	0.0542 (6)	−0.0121 (4)	−0.0169 (4)	−0.0060 (5)

### Geometric parameters (Å, º)

**Table:**

C1—N1	1.3181 (16)	C6—H6B	0.9600
C1—N2	1.3309 (16)	C6—H6C	0.9600
C1—S1	1.7180 (11)	C7—N5	1.3198 (15)
C2—C3	1.3575 (13)	C7—N4	1.3567 (14)
C2—N2	1.3885 (14)	C7—S2	1.6872 (10)
C2—C4	1.4945 (16)	N1—H11	0.79 (2)
C3—C5	1.4570 (14)	N1—H12	0.87 (2)
C3—S1	1.7565 (10)	N2—H2	0.854 (18)
C4—H4A	0.9600	N3—N4	1.3806 (12)
C4—H4B	0.9600	N4—H41	0.943 (17)
C4—H4C	0.9600	N5—H51	0.81 (2)
C5—N3	1.2906 (13)	N5—H52	0.80 (2)
C5—C6	1.4961 (16)	OW1—HW1	0.78 (3)
C6—H6A	0.9600	OW1—HW2	0.71 (3)
			
N1—C1—N2	124.00 (11)	C5—C6—H6C	109.5
N1—C1—S1	124.99 (10)	H6A—C6—H6C	109.5
N2—C1—S1	111.01 (8)	H6B—C6—H6C	109.5
C3—C2—N2	111.36 (9)	N5—C7—N4	117.81 (9)
C3—C2—C4	131.66 (10)	N5—C7—S2	122.67 (8)
N2—C2—C4	116.95 (9)	N4—C7—S2	119.51 (8)
C2—C3—C5	131.75 (10)	C1—N1—H11	118.0 (15)
C2—C3—S1	111.21 (8)	C1—N1—H12	113.9 (14)
C5—C3—S1	116.95 (7)	H11—N1—H12	127.9 (19)
C2—C4—H4A	109.5	C1—N2—C2	115.94 (9)
C2—C4—H4B	109.5	C1—N2—H2	120.9 (11)
H4A—C4—H4B	109.5	C2—N2—H2	123.1 (11)
C2—C4—H4C	109.5	C5—N3—N4	118.97 (9)
H4A—C4—H4C	109.5	C7—N4—N3	118.45 (9)
H4B—C4—H4C	109.5	C7—N4—H41	115.1 (10)
N3—C5—C3	113.92 (9)	N3—N4—H41	126.3 (10)
N3—C5—C6	126.22 (10)	C7—N5—H51	122.4 (14)
C3—C5—C6	119.81 (9)	C7—N5—H52	125.2 (13)
C5—C6—H6A	109.5	H51—N5—H52	112.4 (18)
C5—C6—H6B	109.5	C1—S1—C3	90.43 (5)
H6A—C6—H6B	109.5	HW1—OW1—HW2	106 (3)
			
N2—C2—C3—C5	175.76 (11)	C4—C2—N2—C1	177.27 (11)
C4—C2—C3—C5	−2.4 (2)	C3—C5—N3—N4	177.15 (9)
N2—C2—C3—S1	−0.57 (12)	C6—C5—N3—N4	−0.16 (18)
C4—C2—C3—S1	−178.69 (11)	N5—C7—N4—N3	−0.63 (16)
C2—C3—C5—N3	177.59 (11)	S2—C7—N4—N3	178.17 (8)
S1—C3—C5—N3	−6.25 (13)	C5—N3—N4—C7	−174.48 (10)
C2—C3—C5—C6	−4.92 (19)	N1—C1—S1—C3	177.38 (12)
S1—C3—C5—C6	171.25 (10)	N2—C1—S1—C3	−2.17 (9)
N1—C1—N2—C2	−177.21 (12)	C2—C3—S1—C1	1.55 (9)
S1—C1—N2—C2	2.34 (13)	C5—C3—S1—C1	−175.38 (9)
C3—C2—N2—C1	−1.15 (14)		

### Hydrogen-bond geometry (Å, º)

**Table:**

*D*—H···*A*	*D*—H	H···*A*	*D*···*A*	*D*—H···*A*
O*W*1—H*W*1···Cl1^i^	0.79 (3)	2.45 (3)	3.2298 (13)	171 (3)
N2—H2···Cl1^ii^	0.853 (18)	2.277 (18)	3.0812 (11)	157.2 (15)
O*W*1—H*W*2···Cl1	0.71 (3)	2.50 (3)	3.2111 (14)	178 (3)
N1—H11···Cl1^iii^	0.78 (3)	2.81 (2)	3.2398 (15)	117.0 (18)
N1—H12···O*W*1^iv^	0.87 (2)	1.97 (2)	2.8354 (19)	174 (2)
N4—H41···S2^v^	0.943 (16)	2.686 (16)	3.6223 (11)	172.0 (14)
N5—H51···Cl1^vi^	0.81 (2)	2.54 (2)	3.3243 (13)	164.9 (18)
N5—H52···O*W*1^vii^	0.80 (2)	2.326 (19)	2.9899 (17)	141.2 (19)
N5—H52···N3	0.80 (2)	2.36 (2)	2.6306 (16)	100.6 (15)
C6—H6*C*···N4	0.96	2.48	2.8433 (18)	102
C6—H6*C*···S2^v^	0.96	2.67	3.4923 (15)	143

Symmetry codes: (i) −*x*+1, −*y*, −*z*+1; (ii) *x*+1, *y*, *z*; (iii) −*x*+2, −*y*+1, −*z*+1; (iv) −*x*+1, −*y*+1, −*z*+1; (v) −*x*, −*y*+2, −*z*; (vi) *x*−1, *y*+1, *z*; (vii) *x*, *y*+1, *z*.
